# Esophageal Stenting in Clinical Practice: an Overview

**DOI:** 10.1007/s11938-018-0181-3

**Published:** 2018-03-19

**Authors:** Bram D. Vermeulen, Peter D. Siersema

**Affiliations:** Department of Gastroenterology and Hepatology, Radboud University Medical Center, Geert Grooteplein-Zuid 8 (route 455), 6500 HB Nijmegen, the Netherlands

**Keywords:** Esophageal stents, Esophageal carcinoma, Refractory benign esophageal stricture, Esophago-respiratory fistula, Benign esophageal perforations, Benign esophageal perforations, Stent migration

## Abstract

**Purpose of review:**

Esophageal stents are used in clinical practice for endoscopic treatment of a wide variety of esophageal diseases and conditions. This review provides key principles and a literature update on the utility and limitations of esophageal stenting in clinical practice.

**Recent findings:**

Indications for esophageal stenting can be subdivided into two groups. The first group consists of patients with malignant or benign dysphagia, in which an esophageal stent restores luminal patency. In the past years, temporary stent placement has increasingly been used in the therapeutic management of refractory benign esophageal strictures. When endoscopic repeated bougie dilation and other endoscopic treatment modalities have failed, an esophageal stent could be considered. Based on the literature, a fully covered self-expandable metal stent may be the preferred choice for the treatment of both malignant and benign dysphagia. The second group consists of patients with leakage from the esophageal lumen into the surrounding tissue. Esophageal leakage can be subdivided into three forms, benign esophageal perforations (iatrogenic and spontaneous), anastomotic leakage after reconstructive esophageal surgery, and fistula. In a carefully selected group of patients, a covered esophageal stent may be used for sealing off the leakage, thereby preventing further contamination of the tissue surrounding the defect. The past few years, several validated prediction tools have been developed that may assist clinicians in the selection of patients eligible for esophageal stent placement. Based on retrospective studies and expert opinion, a partially or fully covered self-expandable metal stent may have a role in treatment of esophageal leakage.

**Summary:**

Research do date supports the utilization of esophageal stents for the treatment of malignant or benign dysphagia and esophageal leakage.

## Introduction

The use of an esophageal endoprosthesis was first described in 1845 to treat malignant dysphagia with a tube made out of ivory [1]. Since then, the endoscopic placement of tubes in the esophagus, i.e., stents, has evolved substantially. The first reported clinical trials date back to the 60s when physicians started to systematically investigate esophageal stents in small cohorts to treat malignant dysphagia [2, 3]. Nowadays, esophageal stent placement is utilized for a wide variety of esophageal diseases. Various stent designs, each with different characteristics, are available for clinical use. The characteristics of esophageal stents vary with regard to the following: (1) mechanical properties, such as the material (metal, plastic, or biodegradable); (2) radial and axial forces acting on the esophageal lumen; and (3) the type and design of a cover that surrounds the stent mesh. The implications of the different stent characteristics on clinical outcome are incompletely understood due to the lack of high-quality evidence from randomized clinical trials (RCT) [4, 5]. Table 1 shows a selection of currently available esophageal stents and their relevant characteristics for clinical practice. The most common indication for an esophageal stent remains palliation of malignant dysphagia. However, palliative brachytherapy is proven to be giving better long-term results in relief of dysphagia compared to metal stents in most patients [6••]. Only in patients with a relatively poor prognosis, an esophageal stent seems to be at least equally effective as a palliative treatment compared to brachytherapy [7•]. Furthermore, treatment with chemoradiotherapy (CRT) has significantly improved over the last decades and is increasingly considered as a curative treatment in patients with esophageal cancer [8, 9]. In clinical practice, this has resulted in a decrease of stent placements for relief of malignant dysphagia in developed countries [10•]. On the contrary, in countries where no routine access to CRT exists, palliative stent placement is still performed in the majority of esophageal cancer patients [11].

**Table 1:** Selected overview of currently available esophageal stents and relevant characteristics for clinical practice

Product	Manufacturer	Placement	Material	Diameter stent body (mm)	Length (cm)	Cover
Alimaxx-ES	Merit Medical	OTW	Nitinol	12/14/16/18/22	7/10/12	FC
Choostent	M.I. Tech	OTW	Nitinol	18/20/22/24	6/17	FC
Evolution	Cook	OTW	Nitinol	18/20	8/10/12	FC/PC
HILZO	BCM	TTS/OTW	Nitinol	20/22	10/12/15	FC/PC
Hanarostent	M.I. Tech	TTS/OTW	Nitinol	18/20/22/24	6/12	FC
Niti-S: single-layered	Taewoong Medical	TTS/OTW	Nitinol	16/18/20/22/24	6/8/10/12/14/15	FC/PC
Niti-S: double-layered	Taewoong Medical	OTW	Nitinol	16/18/20/22/24	6/8/10/12/14/15	FC + UC
SX-ELLA-HV	Ella-CS	OTW	Nitinol	18/20	8.5/11/13.5/15	FC
SX-ELLA-BD	Ella-CS	OTW	Biodegradable	18/20/23/25	6/8/10	UC
Ultraflex	Boston Scientific	OTW	Nitinol	18/23	10/12/15	PC
Wallflex	Boston Scientific	OTW	Nitinol	18/23	10/12/15	FC/PC

*TTS* through-the-scope, *OTW* over-the-wire, *mm* millimeter, *cm* centimeter, *FC* fully covered, *PC* partially covered, *UC* uncovered

A relatively novel indication for stent placement is benign dysphagia as a consequence of a refractory benign esophageal stricture (RBES). Benign esophageal strictures (BES) are caused by a wide variety of esophageal conditions. RBESs are defined as an anatomic restriction because of a cicatricial luminal compromise or fibrosis resulting in clinical symptoms of dysphagia in the absence of endoscopic evidence of inflammation. This may occur as the result of the inability to successfully remediate the esophageal narrowing to a diameter of at least 14 mm over five sessions at 2-week intervals [12]. Benign esophagogastric anastomotic strictures are the most common etiology for a RBES. In addition, post-radiation, caustic and post-endoscopic BES are examples of etiologies that are also associated with a high risk of becoming refractory to treatment with repeated esophageal bougie or balloon dilation (EBD) [13, 14]. This treatment is considered to be the first-line treatment for BES. When EBD fails, other treatment options are available to treat the RBES, including esophageal stent placement [15].

Another indication for esophageal stent placement, other than dysphagia, is esophageal leakage. The underlying mechanisms of esophageal leakage can be diverse, but in all situations, an abnormal connection between the esophageal lumen and the surrounding tissue exists. Esophageal leakage often results in life-threatening situations due to contamination of the mediastinum and consequently septic shock. In these situations, the stent is primarily placed to seal the leakage with a covered stent. It aims to prevent further mediastinal contamination. Stent placement for these indications is often accompanied by fasting, nutritional support with a feeding tube, broad spectrum intravenous antibiotics, and drainage of the contaminated collections in the tissue surrounding the esophageal leakage [16–18].

The etiology of esophageal leakage can be subdivided into malignant and benign. Malignant esophageal leakage is the formation of an esophago-respiratory fistula, predominantly due to recurrent esophageal cancer after treatment with CRT or esophagectomy. Benign causes of esophageal leakage can be spontaneous ruptures (i.e., Boerhaave’s syndrome), iatrogenic instrumental perforations of the esophageal lumen, and anastomotic leakage after reconstructive surgery (e.g., esophagectomy or gastrectomy).

In general, a major limitation of esophageal stent placement for all these indications is the association with a relatively high risk of adverse events [10•]. Especially, stent migration may limit the use of esophageal stents in clinical practice. Table 2 shows an overview of adverse events related with esophageal stent placement for different indications. In the past, efforts have been made to prevent stent migration by adopting new stent designs [19] or by anchoring the stent in the esophageal wall [20].

**Table 2:** Overview of adverse events related with esophageal stent placement for different indications

	Indication		
	Malignant dysphagia^10^ *n* = 1017	Benign dysphagia^41^ *n* = 232	Esophageal leakage^41^ *n* = 599
**Serious adverse event (%)**			
Major bleeding	8.0	3.0	1.3
Aspiration pneumonia	5.0	1.3	0.7
Perforation	2.0	1.3	1.0
**Adverse events (%)**			
Retrosternal pain	30.0	4.3	0.5
Reflux symptoms	7.0	2.6	0.5
Recurrent dysphagia Cause:	31.0	29.0	20.0
Stent migration	11.0	24.5	16.5
Tissue in-/overgrowth	14.0	2.2	2.7
Food obstruction	7.0	2.2	1.1

*n* number of patients, % percentage; ^10^ retrospective cohort analysis; ^41^ systematic review and pooled meta-analysis

In this review, we provide key principles of esophageal stenting and we evaluate recently published literature on advances and limitations of stent placement for the majority of indications to manage esophageal disease.

## Utilization and limitations of esophageal stents

### Dysphagia

#### Malignant dysphagia

Esophageal stenting plays a role in the palliative treatment of patients with esophageal cancer or extrinsic compression on the esophageal lumen due to a malignancy (e.g., mediastinal metastases) [21••, 22]. Selection of the optimal palliative approach can be challenging and is dependent on patient- and disease-related factors. The optimal palliative approach is determined by the prognosis of the patient. Results from a RCT, comparing stent placement to palliative single-dose brachytherapy, showed that brachytherapy gave better long-term relief of dysphagia and was associated with fewer complications. Therefore, patients with mild dysphagia and a relatively long life expectancy are best treated with brachytherapy. Stent placement, as initial palliative approach, is reserved for patients with severe dysphagia and a short life expectancy. As a secondary palliative approach, stent placement can be considered for patients with persistent or recurrent tumor growth after brachytherapy [6••]. In 2005, Steyerberg et al. developed a simple prognostic score that may assist clinicians in identifying patients with a poor prognosis [7•]. Factors that predict the prognosis of inoperable esophageal cancer patients are age, gender, tumor length, presence of metastases, and the World Health Organization (WHO) performance score. Results from this study show that this prognostic tool performs well in identifying patients in whom stent placement is at least equivalent to or even better than brachytherapy. One retrospective cohort study shows that the selection of initial palliative management of patients with inoperable esophageal cancer varies widely in daily clinical practice. Moreover, this study shows that the selected palliative approach was not only associated with patient- and disease-related characteristics, but also with center-related factors [23]. This suggests that more therapeutic guidance is warranted in managing these patients, for example by a selected expert panel. A multidisciplinary tumor board meeting may aid in selecting the most optimal treatment modality, and it is currently recommended to discuss all patients with esophageal cancer that require management [24].

The limitations of stent placement for malignant dysphagia are important to consider when selecting stent placement. For example, the previously mentioned RCT [6••] showed a higher incidence of complications in the stent placement group when compared to brachytherapy, mainly due to hemorrhage. Esophageal hemorrhage is one of several complications that are associated with stent placement in general. Pain requiring analgesics after stent placement is the most frequently observed adverse event. One prospective cohort study reported significant pain during the first 2 weeks after stent placement in almost two-thirds of patients with malignant dysphagia [25]. Recently, a large cohort study reported early and late complications related to stent placement for malignant dysphagia in over a thousand patients, during a period of 23 years [10•]. An overview of the adverse events is shown in Table 2. Observed major complications included esophageal perforation (2%), hemorrhage (8%), pneumonia due to aspiration (5%), fever (5%), fistula formation (3%), and pressure necrosis (2%). Minor complications that were reported included post-procedural pain (30%) and gastroesophageal reflux (7%). Another reported complication after stent placement is recurrent dysphagia (in 31% of patients) due to stent migration (11%), tumor in- or overgrowth (14%), or food obstruction (7%). The results are consistent with those of a recently published systematic review [26].

**Fig. 1:**
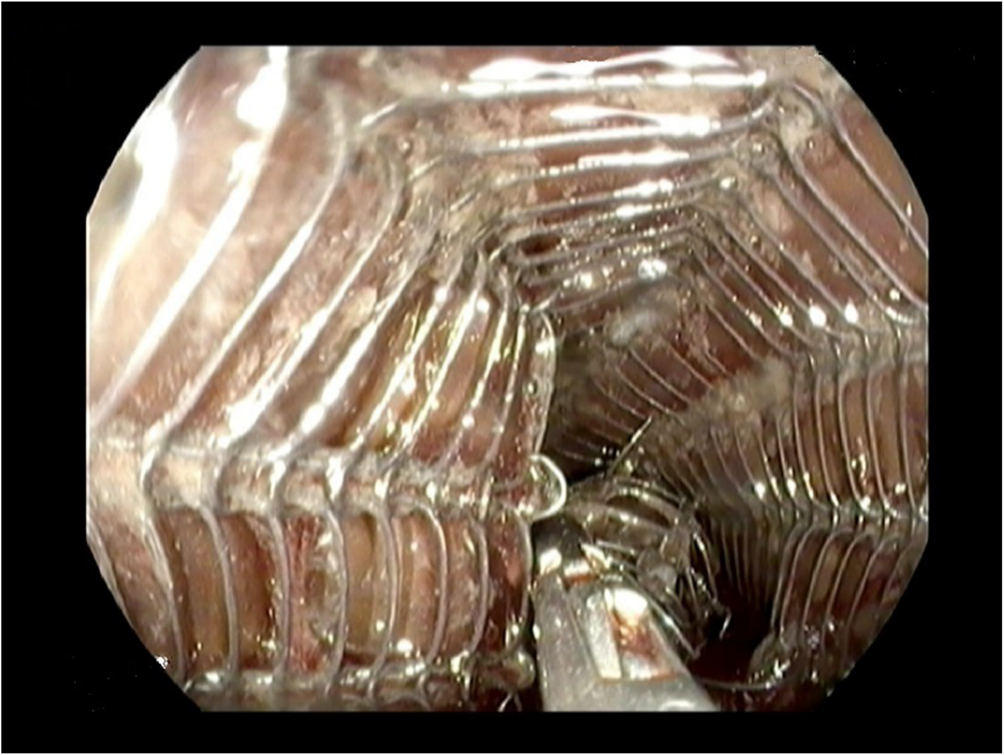
Endoscopic view of the removal of a partially covered SEMS placed for a malignancy. As the patient was complaining of severe retrosternal pain after placement and a perforation was ruled out, it was decided to remove the stent by grasping it with a forceps at the distal end of the stent. SEMS = self-expandable metal stent

The important question remains which type of stent clinicians should use. The latest ESGE guideline on esophageal stenting [21••] recommends to place a fully or partially covered self-expandable metal stents (fcSEMS, pcSEMS) for malignant dysphagia. RCTs have shown that both uncovered SEMSs and covered self-expandable plastic stents (SEPS) are inferior to covered SEMSs due to the higher risk of tumor ingrowth [27] and stent migration, respectively [28]. Recent RCTs have investigated the differences in recurrent dysphagia between pcSEMS and fcSEMS [29•, 30]. No significant differences were found in rates of recurrent dysphagia or any other adverse events after stent placement. However, a recognized disadvantage of a pcSEMS is its possible difficult endoscopic removal. For example, removal may be necessary in case of severe retrosternal pain after stent placement that cannot be relieved with analgesics Fig. 1. Hyperplastic tissue ingrowth at the uncovered stent mesh may lead to embedding into the esophageal mucosa, which prevents immediate endoscopic removal of the pcSEMS. Removal of an embedded pcSEMS can be achieved with the stent-in-stent method [31]. Using this technique, a similarly sized fcSEMS is placed inside the previously placed embedded stent. Over a period of 10 to 14 days, pressure necrosis of the hyperplastic tissue occurs as a result of friction. Hereafter, both stents can mostly easily be removed during a second endoscopic procedure. The stent-in-stent technique may also be applied in patients with recurrent malignant dysphagia due to tumor progression and overgrowth at the proximal or distal stent ends [32]. To treat tumor overgrowth, a second fcSEMS is placed through the first stent, adequately covering the site of tumor overgrowth. Both SEMSs are then left in place as long as sufficient palliation of dysphagia is achieved.

In summary, based on the literature and our expert opinion, the preferred choice is to place fcSEMSs for malignant dysphagia as a first- or second-line palliative treatment.

#### Benign dysphagia

In contrast to malignant strictures, the mainstay in therapeutic management of BES is repeated EBD. The dilating effect of EBD is applied for a short period of time during the endoscopic procedure. It should be recognized that EBD for malignant strictures is associated with a higher risk of esophageal perforation and therefore should be performed cautiously in these patients [33]. When repeated EBD fails to achieve satisfactory relief of benign dysphagia, other endoscopic treatment modalities are available. For example, 4-quadrant corticosteroid injections may be performed in peptic strictures [34] and needle-knife incisions in esophagogastric anastomotic strictures [35]. Esophageal stent placement is considered the last endoscopic treatment option in the management algorithm for RBES [15]. As opposed to EBD, a temporary stent acts as a dilator that is kept in place for a substantially longer time of several weeks. Notorious etiologies of BES, associated with a high risk of becoming refractory to repeated EBD are caustic, radiation-induced, and esophagogastric anastomotic strictures [36–39]. A rising incidence of post-endoscopic strictures is expected as a consequence of new endoscopic interventional treatment modalities in daily clinical practice. For example, in recent guidelines, endoscopic mucosal resection (EMR), endoscopic submucosal dissection (ESD), and radiofrequency ablation (RFA) are recommended to treat premalignant stages and early-stage malignancies in Barrett’s esophagus [40], all carrying a risk of stricture formation [36].

In the past decade, clinical success of temporary stent placement for RBES has been investigated in mainly non-randomized and uncontrolled observational cohort studies. Not so long ago, two systematic reviews were published [41, 42], reporting and comparing all studies that investigated esophageal stents in RBES. Three stent types investigated in predominantly retrospective studies included SEPS, fcSEMS, and most recently biodegradable (BD) stents. In terms of clinical success, defined as the resolution of dysphagia without needing any further intervention at the end of follow-up, all stent types showed similar results and the overall clinical success rate was approximately 40%. In terms of migration rate and risk for other adverse events, no significant differences were observed between the stent types. In general, the average migration rate was 29% and the adverse event rate was 21% for all stent types [42]. An overview of the adverse events related to stent placement for RBES are shown in Table 2. Remarkably, these adverse event rates are comparable to the rates that were observed in stenting of patients with malignant dysphagia. The Polyflex SEPS (Boston Scientific, Natick, Massachusetts) is the only FDA-approved esophageal stent for RBES. In several studies, the Polyflex was proven to be effective for treatment of RBES [43–45], but due to the high risk of stent migration [44] and other adverse events [45], the production of the Polyflex has been terminated.

As for malignant dysphagia, but without formal FDA approval, fcSEMS have also been investigated in clinical trials treating RBES [46–48]. The clinical success rates of fcSEMS are likely to be comparable and not significantly different from SEPS and BDS [42], although this has never been investigated in head-to-head RCTs. Furthermore, temporary placement of a pcSEMS is not recommended because of the higher risk of hyperplastic tissue ingrowth [49]. It is assumed that a longer stent indwell time is associated with a risk of stent embedment. However, different periods of esophageal stenting for RBES have not been compared in studies. The ESGE guideline on esophageal stenting [21] recommends placing a fcSEMS and to keep it in place for at least 6–8 weeks and no more than 12 weeks, to achieve optimal treatment response and reduce the risk of stent embedding. In addition, one retrospective cohort study, investigating the safety of esophageal stenting in RBES patients, found no association between indwell time and risk of adverse events [50].

Lastly, BD stents have been introduced as treatment option in patients with RBES. The Ella BD stent (Ella CS, Hradec Králové, Czech Republic) has been investigated in several clinical studies. An important advantage of BD stents is that no stent removal is indicated due to its biodegradable feature, making it a potentially more cost-effective and patient-friendly treatment option. After 4 months, the BD stent appears to be dissolved in most patients, but the stent increasingly loses radial force after a couple of weeks due to the degradation process. Since the first study in 2010 with the BD stents [51], a few more studies have been performed assessing safety and efficacy [42, 52], all concluding that the BD stent is safe for use in RBES. In addition, the only RCT with BD stents was performed in 2014 in the UK, but was terminated prematurely due to recruitment issues [53]. One prospective cohort study was performed that compared BD stents with other types of esophageal stents, i.e., SEPS and fcSEMS [54]. The authors concluded that placement of BD stents and fcSEMS may lead to long-term relief of dysphagia in 30 and 40% of patients, respectively. The use of a sequential BD stents in patients with RBES was reported by Hirdes et al. [55]. A total of 59 BD stents were placed in 28 patients. Unfortunately, this strategy proved to be effective in only a small proportion of patients. After 6 months, only 15% of patients, who had recurrent dysphagia after the first BD stent placement, were still dysphagia-free after placement of a second BD stent.

In summary, based on the literature and our expert opinion, it seems preferably to place fcSEMS for treatment of RBES, after repeated EBD and other endoscopic treatment modalities have failed to achieve long-term relieve of dysphagia. If available, BD stents may be considered as an effective, patient-friendly alternative for fcSEMS placement, as stent removal is avoided.

### Esophageal leakage

The mediastinum, pleural, and abdominal cavity and airways are structures surrounding the esophagus that may be affected by esophageal leakage. The covered part of a stent is placed over the luminal defect to seal off the leak and possibly limit the contamination of surrounding structures.

#### Malignant esophageal fistula

The formation of fistula connecting the esophageal lumen to surrounding tissue may be the result of predominantly esophageal cancer and less frequently extrinsically growing cancer. End-stage malignant disease for which palliative management is indicated is seen in the majority of patients. Furthermore, when an esophageal fistula is present, concomitant metastatic disease is found in approximately 90% of patients [56]. Fistula formation may also be the result of tumor necrosis induced by CRT.

**Fig. 2:**
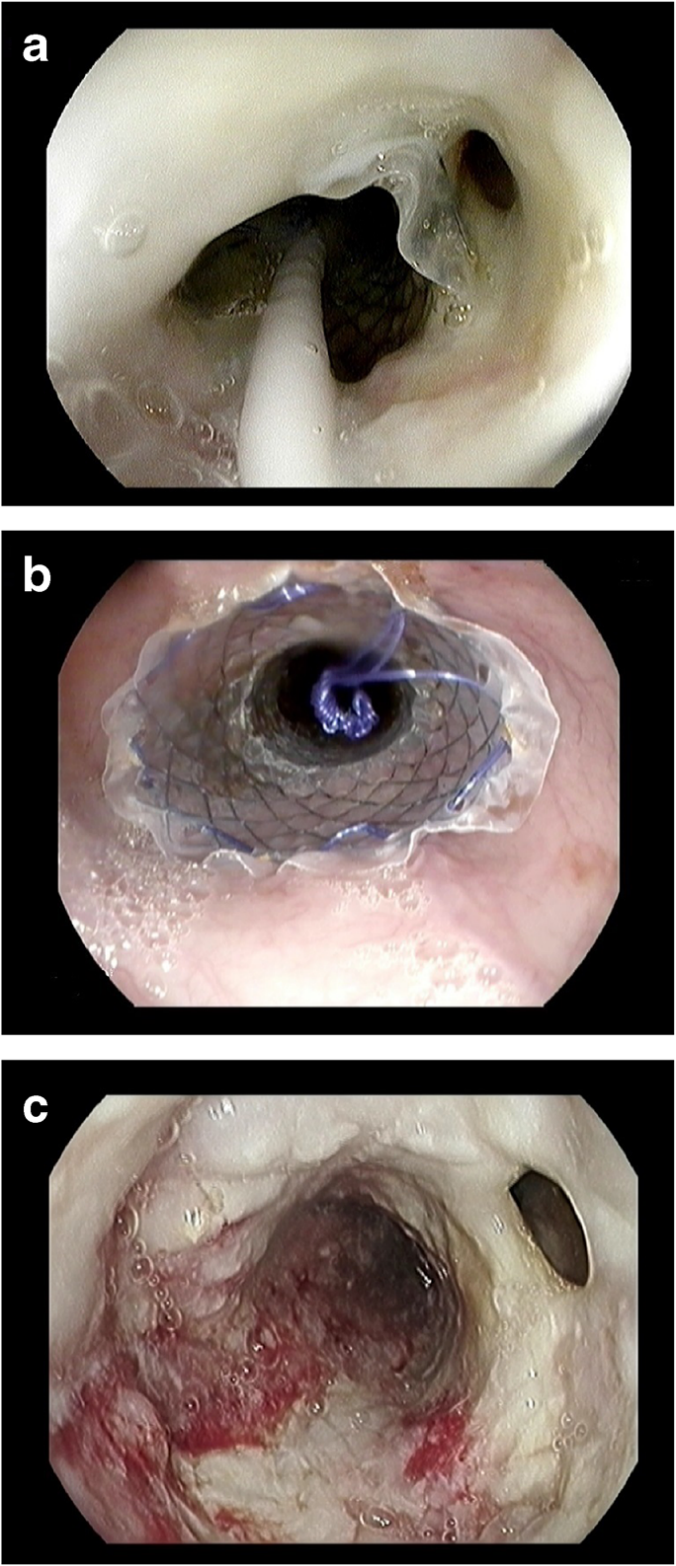
**a** Endoscopic view of patient with an esophago-respiratory fistula and a slightly distally migrated fully covered SEMS. **b** The SEMS was removed and replaced by another type of fully covered SEMS to seal the esophago-respiratory fistula. **c** Unfortunately, the esophago-respiratory fistula had persisted when the SEMS was removed after 8 weeks and a surgical approach was chosen to close the fistula. SEMS = self-expandable metal stent

The management of malignant fistula always includes conservative and may include endoscopic treatment modalities. Conservative management may consist of ‘nil by mouth’, duodenal tube feeding, intravenous antibiotics, and adequate drainage of contaminated cavities surrounding the fistula. Endoscopic management with a pcSEMS or fcSEMS to seal esophageal fistula and palliate concomitant dysphagia has been shown to be feasible and effective. In recent studies, few retrospective case series have been published of patients treated with esophageal stents for malignant fistula [57–59••]. One prospective cohort study included 15 patients that were treated with pcSEMS or fcSEMS for esophageal fistula [60•]. The authors demonstrated successful restoring of luminal patency in all patients. The fistulae were successfully sealed off in all but one patient (93%) Fig. 2. In four patients, stent migration was observed and subsequently sealed off with placement of a second stent.

Until now, no randomized studies have compared pcSEMS or fcSEMS for sealing off esophageal fistula. Based on available literature and the high risk of concomitant malignant dysphagia, a fcSEMS seems the preferred palliative choice for endoscopic management of malignant fistula.

#### Benign esophageal perforations

Benign esophageal perforations (BEP) can be subdivided into spontaneous esophageal perforations, also known as Boerhaave’s syndrome (BS) and iatrogenic esophageal perforations (IEP).

BS is associated with excessive vomiting leading to a barogenic trauma in the distal esophagus. Patients with BS typically present with vomiting followed by pain, dyspnea, and septic shock [17]. Early diagnosis, within 24 h, is crucial for successful outcome and can be challenging in patients with BS because the symptom onset usually sets off in an out of hospital setting [18, 61]. Esophageal stent placement to treat BS has been shown to be feasible and may have a role in the treatment algorithm [62, 63].

IEP induced by instrumentation during endoscopy is an emerging entity, as more invasive endoscopic treatment options (e.g., EMR and ESD) are being performed [64–66]. EBD for esophageal narrowing and pneumatic dilation for achalasia is associated with a relatively high risk of perforation [67, 68]. IEP is managed according to a similar treatment algorithm as in patients with BS [17], but associated with a better prognosis because of in-hospital symptom onset, likely resulting in an early diagnosis within 24 h.

In clinical practice, therapeutic management of BS and IEP is comparable, as available treatment strategies are conservative, endoscopic, or surgical in both groups [61, 69]. High-quality evidence from RCTs on therapeutic management is lacking due to the rarity of BEP in daily practice [70]. Therefore, evidence on treatment decisions is solely based on retrospective data. In 2009, Abbas et al. developed a clinical tool, the Pittsburgh perforation severity score (PSS), to predict patients’ prognosis following BEP [71]. After validation, the PSS proved a reliable tool to stratify patients into risk groups with differential morbidity and mortality outcomes [72•]. However, further prospective research is warranted to evaluate utility of this tool in clinical decision-making. With respect to the incidence of BEP, a nation-wide or international prospective registry seems the most appropriate study design to generate evidence for the optimal therapeutic management of BEP.

In the past decade, a rise in esophageal stent placement to manage BEP is observed. In the USA, the use of esophageal stents for BEP has increased from 7% in 2007 to 30% in 2014 [73]. In the UK, a similar trend is observed in a nation-wide cohort analysis [70]. In a newly proposed BEP treatment algorithm, based on the PSS, stenting is incorporated [72•]. According to this treatment algorithm, esophageal stents may have role in patients stratified in the low and medium risk groups. In these patients, BEP is accompanied by a non-contained leak into the surrounding structures. A covered stent, followed by adequate drainage of the fluid collections, is expected to cease further contamination by sealing off the leak.

The clinical success of stent placement for BEP, defined as successful closure of the perforation after single of sequential stent placement, varies widely from 50 to 86% [41, 62, 74]. Very recently, Van Halsema et al. developed a prediction rule for successful stent placement for esophageal anastomotic leakage, perforations, and fistula [59••]. The prediction rule consisted of four clinical predictors: etiology, location and size of the leak, and CRP level at diagnosis. After validation in a different patient cohort, the rule was found to significantly discriminate between failure and success of stent placement in patients with a predicted low (≤ 50%) or high (≥ 70%) clinical success. Therefore, this clinical tool may be supportive in clinical decision-making and informing patients with various types of benign perforations, leakage, and fistula.

The selection of the right stent design in treatment of BEP also remains a challenge, as no high-quality evidence is available. In a pooled meta-analysis of several case series investigating stent placement for BEP, stent migration was reported in around 20% of patients [41]. An overview of most prevalent adverse events related to stent placement for BEP is shown in Table 2. In BEP, the consequence of stent migration may have a higher impact on the clinical outcome due to inadequate sealing of the defect, leading to further contamination of the surrounding tissue. Prevention of stent migration is therefore of major importance. Covered stent designs with a wider diameter may result in fewer stent migrations. One retrospective case series investigated a large diameter (stent body: 24 mm, flares: 32 mm) fcSEMS in 34 patients with BEP, anastomotic leakage, and fistula [57]. Disappointingly, stent migration was observed in 41% of patients.

In summary, prediction rules may aid in careful selection of patients that could benefit from stent placement. Based on the literature and our expert opinion, it seems preferable to place a pcSEMS or fcSEMS in selected patients. Furthermore, we hypothesize that placing a pcSEMS may contribute to even better sealing of the perforation and reducing the risk of stent migration, as a result of stent embedding of the uncovered stent mesh ends in the esophageal mucosa.

#### Preventing stent migration

Ever since the use of esophageal stents in clinical practice, the most important drawback of stenting remains stent migration, occurring in approximately one-third of all patients [10•, 29•, 42]. In the past few years, various attempts were done to investigate methods to prevent stent migration by anchoring the stent to the esophageal wall. An interesting method to anchor the stent is the placement of through-the-scope (TTS) clips [75] or over-the-scope (OTS) clipping devices [76]. The clip is placed over the proximal stent mesh end, into the esophageal wall. Retrospective case series show promising results, particularly for OTS clipping, but are still reporting migration ranging from 13 to 17% [20, 75, 77].

Another promising method of anchoring is endoscopic suturing with the novel, FDA-approved, endoscopic suturing device [78]. A systematic review and meta-analysis was performed to analyze retrospective case series (*n* = 14), investigating endoscopic suture fixation of esophageal covered SEMS [79•]. Results from some case series look promising, but pooled data analysis revealed that stent migration occurred in still 1 of 6 patients after suture fixation. Needless to say that high-quality evidence provided by comparative RCTs is required.

## Conclusion

The endoscopic placement of esophageal stents remains an important treatment option in palliative care of malignant dysphagia. New indications for esophageal stenting have, however, emerged. Temporary stent placement is found to be effective for refractory benign esophageal strictures and for esophageal leakage, which includes spontaneous (Boerhaave’s syndrome) and iatrogenic perforations, anastomotic leakage, and fistula. However, high-quality evidence comparing esophageal stenting with other available treatment options is lacking. Furthermore, esophageal stenting is associated with a substantial risk of adverse events (e.g., stent migration, recurrent dysphagia, retrosternal pain). Therefore, careful selection of patients on an individual level is recommended when considering esophageal stent placement.
